# Feasibility of MV CBCT‐based treatment planning for urgent radiation therapy: dosimetric accuracy of MV CBCT‐based dose calculations

**DOI:** 10.1120/jacmp.v16i6.5625

**Published:** 2015-11-08

**Authors:** Mareike Held, Penny K. Sneed, Shannon E. Fogh, Jean Pouliot, Olivier Morin

**Affiliations:** ^1^ Department of Radiation Oncology University of California San Francisco San Francisco CA USA

**Keywords:** dose calculation, emergency treatments, MV CBCT, radiation therapy, treatment techniques

## Abstract

Unlike scheduled radiotherapy treatments, treatment planning time and resources are limited for emergency treatments. Consequently, plans are often simple 2D image‐based treatments that lag behind technical capabilities available for nonurgent radiotherapy. We have developed a novel integrated urgent workflow that uses onboard MV CBCT imaging for patient simulation to improve planning accuracy and reduce the total time for urgent treatments. This study evaluates both MV CBCT dose planning accuracy and novel urgent workflow feasibility for a variety of anatomic sites. We sought to limit local mean dose differences to less than 5% compared to conventional CT simulation. To improve dose calculation accuracy, we created separate Hounsfield unit–to–density calibration curves for regular and extended field‐of‐view (FOV) MV CBCTs. We evaluated dose calculation accuracy on phantoms and four clinical anatomical sites (brain, thorax/spine, pelvis, and extremities). Plans were created for each case and dose was calculated on both the CT and MV CBCT. All steps (simulation, planning, setup verification, QA, and dose delivery) were performed in one 30 min session using phantoms. The monitor units (MU) for each plan were compared and dose distribution agreement was evaluated using mean dose difference over the entire volume and gamma index on the central 2D axial plane. All whole‐brain dose distributions gave gamma passing rates higher than 95% for 2%/2 mm criteria, and pelvic sites ranged between 90% and 98% for 3%/3 mm criteria. However, thoracic spine treatments produced gamma passing rates as low as 47% for 3%/3 mm criteria. Our novel MV CBCT‐based dose planning and delivery approach was feasible and time‐efficient for the majority of cases. Limited MV CBCT FOV precluded workflow use for pelvic sites of larger patients and resulted in image clearance issues when tumor position was far off midline. The agreement of calculated MU on CT and MV CBCT was acceptable for all treatment sites.

PACS numbers: 87.55.D‐, 87.57.Q‐

## I. INTRODUCTION

Cone‐beam CT acquisition has become a routine procedure in radiation oncology. It provides a 3D image of the patient in treatment position immediately before treatment and is regularly used as a method to increase precision of patient alignment. Recently, there has been a growing interest in using cone‐beam CT images for other purposes, such as dose calculations and monitoring of patient anatomy, during the course of treatment. Dose calculations using MV CBCT images are feasible.^1^, ^2^ However, consistently converting CT numbers to electron density can be difficult.^3^, ^4^, ^5^, ^6^ Previous research studies reported a gamma index of 98% for 3% and 3 mm criteria that described dose calculation accuracy on MV CBCT for head‐and‐neck cases, using in‐house developed image correction filters.^(7)^ However, this was based on a limited patient population and dose calculations needed verification in more anatomical sites to be clinically relevant. Aubry et al.^(8)^ presented similar results for phantom studies of the pelvic region. These research studies focused on dose calculation to improve the accuracy for image‐guided radiation therapy (IGRT). Other applications include emergency treatments using CBCT‐ and MV CT‐based planning to reduce patient setup and waiting time.^9^, ^10^ Here, similar results to our initial experience with head‐and‐neck cases^(7)^ have been verified for commercially available systems. Furthermore, MV CBCT‐based dose calculation was investigated for different anatomical sites using a lateral flat‐panel offset for an extended FOV.

MV CBCT imaging has advanced considerably, providing better image quality while still lowering dose to the patient. The most recent commercially available systems are considered second‐generation MV CBCT systems. This study combines both approaches mentioned above: reduce patient setup and planning time while still providing accurate dose calculation that considers inhomogeneity corrections.

The objective of this study was to investigate the feasibility of emergency radiotherapy treatment (ERT) dose planning on selected anatomical sites based on MV CBCT images acquired on a commercial treatment machine.

## II. MATERIALS AND METHODS

### A. ERT workflow

**Figure 1:**
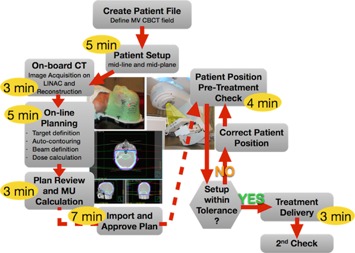
ERT workflow. Flowchart to describe the workflow for combined simulation, planning and treatment of emergency patients in one session with the expected times for each step.

A streamlined workflow was developed to reduce the amount of time the patient spends from the time a decision is made to undergo emergency radiation therapy to the delivery of the treatment. The workflow combined simulation, planning, QA, and treatment into a single session so that the patient is set up once on the treatment machine and remains on the linac couch until after treatment delivery. Ideally, this would be done within a 30 min time slot to avoid delaying other patients. Figure 1 outlines the suggested workflow. The goal was to automate the process as much as possible by using scripting within the planning software to quickly produce a simple treatment plan, and by merging each person's responsibilities into one step.

Dose calculation accuracy on MV CBCT images was the other focus of this study. First, phantom studies were performed to determine the reconstructed intensity for different tissue densities. This resulted in two different calibration curves, which were added to a commercial planning system. Then, simple dose calculations on phantom and patient images were performed. The plans included clinically relevant examples of emergency and palliative treatments. All MV CBCT based treatment plans were compared to the simulation CT‐based plans as the gold standard and were displayed in percent difference relative to the simulation CT. All CT were acquired on a Philips MX8000 CT scanner (Philips Healthcare, Andover, MA).

### B. MV CBCT imaging

The cone‐beam images used in this study were acquired on the Siemens Artiste and the In‐Line kView cone‐beam system (Siemens, Munich, Germany). This commercially available in‐room imaging system was integrated into the treatment machine. It used a low‐energy treatment beam of 1 MV with a carbon target and no flattening filter. More detailed information on the system has been described in the literature.^11^, ^12^, ^13^ The system includes two available imaging modes: regular field of view (FOV), and extended FOV. The In‐Line kView MV CBCT system acquired images with a FOV of approximately $27\times 27\times 27{\text{cm}}^{3}$ when using the regular FOV mode. It acquired images at 1° increments for a 200° rotation around the patient. The FOV for MV CBCT systems was one of the main limitations. To perform dose calculation, the entire anatomy had to be captured along the beam path. Furthermore, truncating the anatomy caused missing data artifacts.^(4)^ To obtain a larger FOV, two approaches have been previously reported. The first approach fuses the kV CT reference image with the MV CBCT.^14^, ^15^ Alternatively, the FOV can be extended during the image acquisition.^(1)^ The aim of this study was to perform simple dose planning without the acquisition of a CT image. Thus, a lateral flat‐panel offset of 5.5 cm and a full rotation around the patient was used to capture larger anatomy, such as the chest or the pelvis. This allowed for an extended FOV up to $31\times 31\times 27{\text{cm}}^{3}$. Due to the uneven beam profile, the image corrections varied based on which imaging mode was used. The system applied binning, averaging, and diffusion filters automatically to reduce image artifacts, such as cupping artifacts, ring artifacts, and noise.^(16)^ The exposure used during an image acquisition was measured in monitor units (MU) and could be as low as 1 MU. The exposure range of interest for this study, however, was between 3 and 15 MU (equivalent to about 3−15 cGy), depending on the treatment site, to improve image quality.

### C. Electron density calibration

Most treatment planning systems use a lookup table that converts the reconstructed gray‐level intensity of each CT image voxel into a physical or electron density value, thereby mapping the tissue inhomogeneity of the imaged object. Consequently, the reconstructed intensity of tissues has to remain constant over space and time in order to perform dose calculation accurately. Due to the energy dependence of X‐ray interaction with matter,^17^, ^18^ the reconstructed intensity‐to‐physical‐density calibration for fan‐beam CT scanners was invalid for MV CBCT images. Thus, the MV CBCT system had to be calibrated independently. Two water phantoms with density inserts were used to calibrate the Hounsfield units (HU) to physical density for both the regular FOV (rFOV) and extended FOV (eFOV). Phantom 1, used to calibrate the rFOV, was a commercially available head‐sized cylindrical container filled with water. The phantom dimensions were 17 cm in diameter and 25 cm in length. A similar phantom was used for MV CBCT dose calibration in previous studies.^4^, ^7^ A plastic disc with cutouts was glued inside the center to hold seven different density inserts (air, lung inhale, lung exhale, adipose, water, trabecular bone, and dense bone) (CIRS Inc. model 062, Norfolk, VA) in place. Phantom 2, used to simulate the dimensions of a pelvis patient, measured approximately $38\times 25\times 25{\text{cm}}^{3}$ and was used to calibrate the eFOV. To avoid sharp edges that could cause unnatural artifacts during imaging, acrylic sheets were bent under heat to form a human‐like pelvis object. The inserts of phantom 1 are routinely used for clinical HU calibration of the departmental CT scanner. Thus, the physical and electron density were well known. For phantom 2, customized inserts made from epoxy 670 HT (Reynolds Advanced Materials, Brighton, MA) were glued to the inside. Each insert contained between 10% and 30% of ${\text{CaCo}}_{3}$ to vary its density.

Both phantoms were imaged on both the standard kV CT and the MV CBCT. A region of interest (ROI) was defined across several center slices for each insert. The MV CBCT calibration curve was obtained by plotting the average HU of each ROI against the respective physical density. Afterwards, the value for air was adjusted slightly based on ROIs within air cavities of patient MV CBCT images. The original MV CBCT curves were extrapolated linearly to 0 density and extended to the origin of the plot, as required by the planning software.

### D. CT and MV CBCT dose planning on phantom images

To determine the feasibility of accurate dose calculation on CBCT images using commercially available software, dose predictions were compared on phantom CT and CBCT images. All treatments were planned using the treatment planning software Pinnacle 9.2 (Philips Healthcare, Andover, MA), which is our clinical standard planning system for treatments on the Siemens Artiste. Each CT and CBCT image pair was rigidly aligned prior to treatment planning. A new patient file was created for each image set. During the dose planning process, the density calibration was chosen accordingly to the imaging mode that was used during the acquisition of that specific image series (CT or rFOV MV CBCT or eFOV MV CBCT). Single beams with field sizes between 5 cm×5 cm and 10 cm×10 cm were applied to each CT and the plan was copied to the MV CBCT. It was verified that the isocenter, dose grid, beam, and other parameters were identical for each CT and the matching MV CBCT. Once the dose distribution was calculated, the 3D dose of each CT and MV CBCT pair was exported and compared using MATLAB (MathWorks, Natick, MA).

### E. CT and MV CBCT dose planning on patient images

Over a period of several months, patient cone‐beam CT images were collected to build a case library based on clinical cases. For dose comparison purposes, the MV CBCT images were rigidly aligned to the patient's planning CT. Each scan was associated with a new patient in the planning system. A simple emergency treatment plan was created on the CT and copied to the aligned MV CBCT with two different prescriptions. Prescription 1 prescribed the treatment dose to a point at mid‐plane to find the percentage difference of MU. Prescription 2 was set to 100 MU per plan. The dose distribution was analyzed in MATLAB, using a voxel‐by‐voxel comparison. The mean dose difference and standard deviation (SD) were determined for dose regions of 20% and more of the prescription dose. Additionally, the dose distribution was analyzed using the gamma index criterion for 2D axial plane in the SNC patient software (Sun Nuclear, Melbourne, FL). The various sites imaged during this period included seven brain, nine thoracic, and nine pelvis cases, as well as two extremities, one knee, and one foot.

## III. RESULTS

### A. ERT workflow feasibility

Prior to clinical implementation, several test runs were performed on phantoms. The general workflow required to first register the patient/phantom in the patient‐management software (MOSAIQ; Elekta AB, Stockholm, Sweden). A diagnosis, prescription, site setup, and MV CBCT treatment field were entered before the patient arrived. Once the patient/phantom was set up on the treatment machine, the MV CBCT was acquired. The reconstructed image was sent to the treatment planning software. Three scripts were used to define the desired treatment plan and export the fields to verify the MU calculation. These scripts were treatment site specific. The only action required during planning was the field definition and adjustment of the prescribed dose. The QA process involved an independent MU calculation using RadCalc (LifeLine Software Inc., Austin, TX). Once the plan was checked and approved, it was imported into MOSAIQ, from where it was forwarded to the treatment machine. The correct patient setup was confirmed using portal imaging immediately before the treatment delivery and the patient position was corrected where necessary. These workflow tests on phantoms showed that the simulation, treatment planning, QA, and delivery could be achieved within 30 min of machine time. The average time for each step is shown in yellow in Fig. 1. Additionally, it required about 5 min to register the phantom and set up the prescription and MV CBCT field before the phantom was set up for treatment. Another 20–30 min were spent on the second check of the treatment plan, uploading of all documents, and billing.

### B. Density calibration

**Figure 2:**
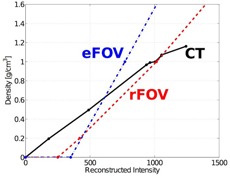
HU calibration. Reconstructed intensity‐to‐density calibration curves for standard CT (solid black), regular FOV (dashed red), and extended FOV (dash‐dot blue) MV CBCT. The MV CBCT curves were extended to an intensity of 0, as required by the planning system.

Figure 2 shows the reconstructed intensity‐to‐physical‐density calibration curves for standard CT and MV CBCT, the latter using regular field of view and extended field of view. They were entered into the planning software to convert the reconstructed intensity into density values. These calibrations were used for all of the study treatment plans, according to the image mode used for acquisition. The stability of the MV CBCT system was validated previously by Morin et al.^(16)^ It was also verified for this specific system over a three‐month period during which Hounsfield units and image quality (noise, contrast‐to‐noise ratio) remained stable.

### C. Dose difference for CT and MV CBCT dose planning on phantoms

**Figure 3:**
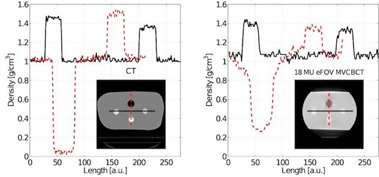
CT and MV CBCT image comparison. Reconstructed density values along a horizontal (solid black) and vertical (dashed red) profile on a CT and extended FOV MV CBCT of a pelvis‐sized water phantom are shown. The phantom included inserts of air and bony materials. The inset images show the standard CT and the eFOV MV CBCT. The sides on the cone‐beam image were truncated due to a limited FOV.

Figure 3 displays CT versus extended FOV MV CBCT horizontal and vertical density profiles in an image slice of the pelvis‐sized water phantom using the calibrations shown in Fig. 2. The eFOV density calibration curve was used to convert the reconstructed intensity into density values. While water was displayed with a density of 1 for both image modalities, the air insert on MV CBCT appears to have a higher density of around 0.28 than on CT, where it was close to 0. Contrarily, the density values for the other inserts appear less dense than on CT. On MV CBCT the densities for the inserts display as 1.40, 1.35, and $1.33\;g/cm^{3}$ compared to CT densities of 1.46, 1.50, and $1.36\;g/cm^{3}$. The comparison of the profiles also shows that the transition between different densities was not as distinct on MV CBCT as it was on CT and that the MV CBCT profiles show more noise compared to the CT image.

**Figure 4:**
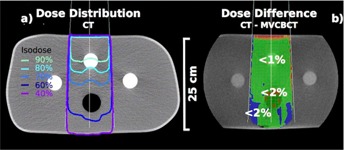
Dose plan comparison in a phantom: (a) dose distribution from one open‐field beam as planned on a CT of a pelvis‐sized water phantom with air and density inserts; (b) percentage dose difference on an eFOV MV CBCT of 18 MU exposure for the same plan relative to the CT‐based planned dose.

The same phantom and intensity‐to‐density conversion was used to compare dose calculation accuracy, ultimately studying dose calculation errors from inaccurate density conversion. Figure 4 compares the dose distribution for a single beam calculated based on CT and MV CBCT for the same phantom as above, but on a different image slice. On the left, Fig. 4(a) shows the planned dose distribution within the phantom. The low‐density region (black circle) displays the air insert in the phantom. Figure 4(b) on the right shows the planned dose difference on the eFOV MV CBCT in percent, relative to the CT planned dose. The percentage difference was less than 2% in most of the beam field. The surface region (0.5 cm) of the phantom showed dose differences around 5%.

### D. Clinical patient cases

#### D.1 Whole brain

**Figure 5:**
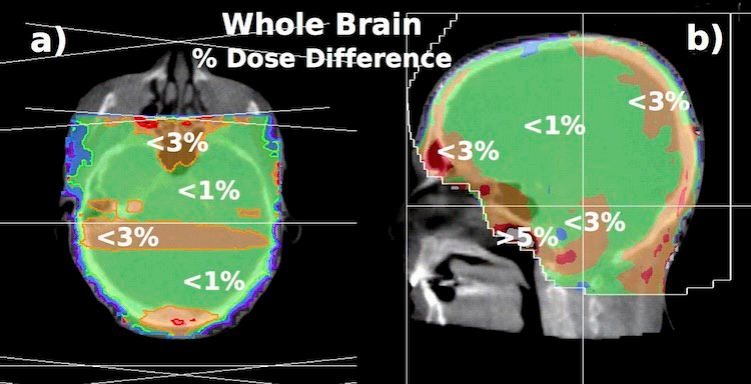
Dose difference for a whole‐brain plan: (a) axial view and (b) sagittal view of the relative dose difference of a whole‐brain treatment with opposed lateral beams. The displayed dose difference is relative to the CT planned dose distribution. The plan used MLCs to shape the PTV.

Figure 5 maps the dose difference between a whole‐brain treatment planned on CT and a 4.5 MU MV CBCT using the rFOV. Figure 5(a) shows a transverse view of the patient's MV CBCT. For prescription 2, the dose difference was less than 3% everywhere except in the nasal cavity, where the dose difference was more than 5%. Similarly, Fig. 5(b) shows a sagittal center slice of the patient with the dose difference distribution less than 1% in the center of the treatment field and less than 3% everywhere else in the brain, except for the nasal air cavity. Prescription 1 showed 1.2% less MU calculated for 300 cGy at a mid‐plane calculation point.

Differences in MU calculated for each plan varied between 0% and 1.2% to achieve the prescription dose of 300 cGy at mid‐plane. Mean dose differences for all studied brain treatments were within 3%. Some cases showed local dose differences higher than 5% in the nasal air cavities. The gamma index for a 2D axial plane was 95% or more for 2% and 2 mm criteria in the SNC patient software.

#### D.2 Thoracic spine treatment

**Figure 6:**
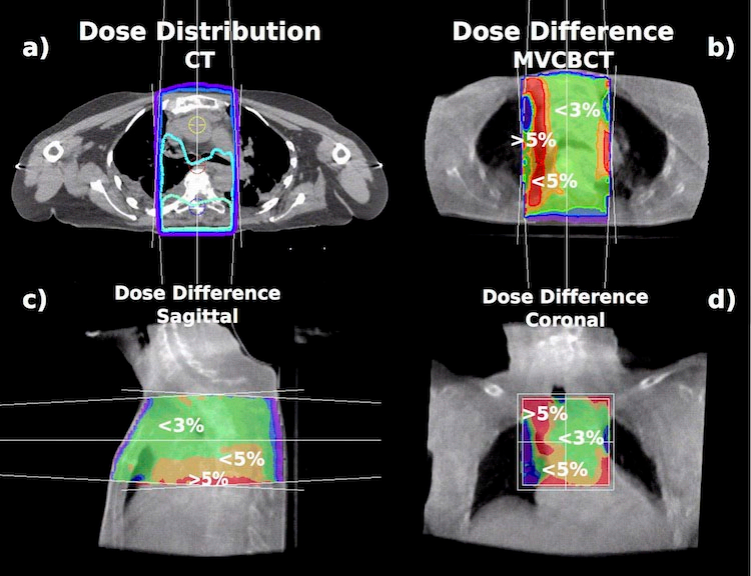
Dose difference for a thoracic spine plan: (a) dose distribution of a thoracic spine treatment with opposed AP/PA beams, planned on a standard CT. Relative dose difference planned on a 13.5 MU exposure eFOV MV CBCT in the transverse (b), sagittal (c), and coronal (d) views.

Figure 6(a) shows the dose distribution of a thoracic spine treatment with opposed AP/PA beams on a CT. The relative dose difference between the CT and the 13.5 MU eFOV MV CBCT was calculated and is presented in Figs. 6(b) through 6(d) (transverse, sagittal, and coronal views, respectively). The local dose difference in soft tissue was less than 3% and up to 5% in some areas close to lung tissue. Dose differences of more than 5% were visible within and beyond lung tissue. The MU for this treatment and a prescribed dose of 700 cGy to mid‐plane were 1.4% lower when calculated on the MV CBCT compared to the CT.

For three out of seven thoracic spine treatments, the gamma index was above 97% for 3% and 3 mm criteria. Two treatments had dose calculation accuracy with a gamma index of 100% for increased criteria of 5% and 3 mm. Locally, the gamma index failed the given criteria within lung tissue. Large dose differences between the CT and MV CBCT also occurred within the 5 mm buildup region near the skin. The difference of MU calculated on CT and MV CBCT for a prescribed dose of 700 cGy to mid‐plane using two opposed beams ranged between 1.4% and 3.6%.

#### D.3 Pelvis treatment

**Figure 7:**
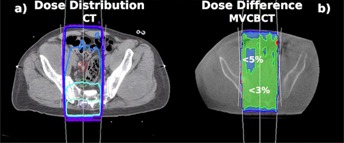
Dose difference for a pelvis plan: (a) the CT planned dose distribution and (b) relative dose difference planned on a 13.5 MU exposure eFOV MV CBCT of a pelvis patient. The treatment plan consisted of opposed AP/PA, with a one‐third to two‐thirds beam weighting.

Figure 7 presents the dose distribution of opposed AP/PA beams in a pelvis (a) and the relative dose difference in percent of the same plan calculated based on an eFOV MV CBCT (b). The dose difference resulting from this comparison was less than 3% in about two‐thirds of the volume. The dose difference was less than 5% in the bowel area, which contains air cavities. Due to the time difference of the image acquisition, the size and location of the air cavities were not identical. The dose calculated based on the MV CBCT was underestimated in the posterior skin area where the dose difference was about 5%. The number of MU calculated on the CT and the MV CBCT were the same for a prescribed dose to mid‐plane.

For all studied simple pelvis treatments, the gamma index was between 90% and 98% for 3% and 3 mm criteria. Local dose discrepancies that failed the gamma index were within air cavities and, in some cases, within the 5 mm buildup region near the skin surface. For a prescription point at mid‐plane, MU were calculated within −1% and +1.9% accuracy for MV CBCT‐based images.

#### D.4 Extremities — knee treatment

**Figure 8:**
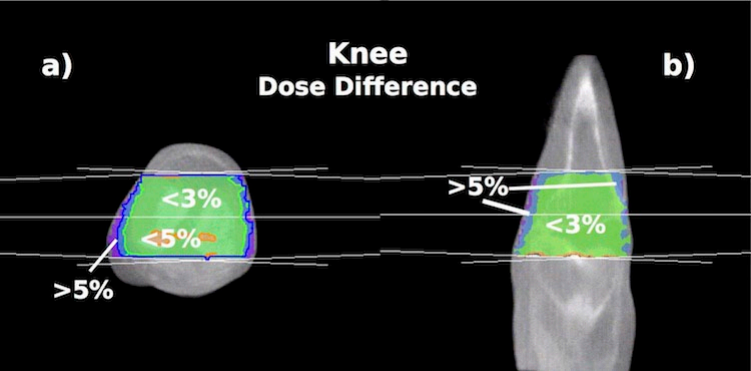
Dose difference for a knee plan: (a) transverse and (b) coronal views of a knee showing the dose difference relative to a standard CT planned on a 4.5 MU exposure rFOV MV CBCT. Opposed lateral beams with equal weighting were used in this plan.

The dose difference between the same CT and MV CBCT treatment plan for a knee is presented in Fig. 8. It shows the transverse (a) and coronal (b) views of a 4.5 MU exposure rFOV MV CBCT. The plan consisted of opposed lateral beams. The dose differences were less than 3% in most areas. The transverse view shows a small area within the knee in which the MV CBCT dose was overestimated up to 5%. The dose within 0.5 cm of the lateral sides of the knee was underestimated on the MV CBCT by more than 5%. The MUs obtained from the MV CBCT were 2.2% lower than those from the CT for a dose of 700 cGy at mid‐plane using opposed lateral beams.

All patient case results are summarized in Table 1.

**Table 1:** The range of calculated dose plan accuracies on MV CBCT for each treatment site is shown. The number in parentheses in the left column indicates the quantity of patient cases in each group

*Treatment Site*	*Difference in MU* ($MU_{CT}/MU_{CBCT}$)	*Gamma Index* (3%/3 mm)
Brain (7)	≤1.2%	99%−100%
Thorax (9)	≤1.4%	47%−100%
Pelvis (9)	≤1.9%	90%−99%
Extremity (2)	≤2.2%	92% and 99%

## IV. DISCUSSION

### A. Workflow

To accomplish the entire workflow within 30 min, it required well‐trained staff, good communication, and focus of the entire group. It was key that, prior to the 30 min machine time, the patient was registered in the system, the prescription was entered, and the MV CBCT was set up. The most critical and time‐consuming part was the plan import into MOSAIQ. Also crucial for a smooth workflow was the physician's presence to review and approve the portal images for patient verification immediately before treatment.

The fact that this workflow requires a minimum of 30 min uninterrupted machine time was seen skeptically at first by all the staff involved. However, the benefits outweighed these concerns, one of which is the guarantee of treatment within 30 min after the patient is set up on the treatment couch, minimizing patient waiting times. In the previous setting, urgent‐treatment patients often spent several hours in our department. If a CT was required, available time on the CT scanner for patient simulation rarely aligned with free time on the treatment machine, causing delays and long waiting times for the patient. The entire process was highly disruptive for the physician, physicist, and dosimetrist, who had to be available based on the schedule of two machines. The advantage of the streamlined workflow is to be able to schedule the patient on the treatment machine so that everyone involved knows when his or her work is required. Furthermore, this workflow minimizes patient setup. Especially in cases of heterotopic bone or spine compression treatments, the movement from the bed onto the treatment couch can be extremely painful and difficult for the patient and should be avoided if possible.

In the past, an alternative treatment approach for urgent radiotherapy has been to calculate the dose by hand, solely based on physical patient measurements instead of CT images. The target was defined using 2D portal images. However, physicians are becoming increasingly reluctant to treat patients without sufficient image guidance so as to avoid delivering high doses to normal tissues surrounding the target. The suggested new workflow allows us to view a 3D treatment plan of equal or better quality than from treatment planning without CT simulation.

### B. Extended field of view CBCT

The lateral flat‐panel offset presented several challenges. Due to the uneven beam profile and energy fluence, the reconstructed intensity of an image changed, based on whether the image was acquired with the regular FOV or the extended FOV. During the course of this study, rFOV and eFOV MV CBCT calibration curves were developed independently of each other. The MV CBCT calibration curves were linear due to the energy to attenuation coefficient relation in the MV range.

### C. Dose calculation accuracy for phantoms

MV CBCT of differently sized water and anthropomorphic phantoms were used for initial dose planning. The results show that dose predictions on MV CBCT were within 3% mean difference relative to plans based on standard CT for all tested phantoms. Dose differences can be up to 5% locally within 0.5 cm of the phantom surface facing the beam. The phantoms do not deform, thus image alignment was accurate. The homogeneous dose difference distribution for the water phantoms showed that the HU calibration for water was accurate in rFOV and eFOV for the measured calibration curves and that image nonuniformity was negligible. Although density differences for individual inserts can be observed based on the location within the phantom, this did not prove to cause notable dose calculation inaccuracies.

### D. Considerations for choosing the MV CBCT protocol

Certain cone‐beam properties, as well as properties of the imaged object, can negatively affect image quality. The need for different calibration curves based on the imaging modality showed that the values for reconstructed intensities were not as uniform as for kV CT but rather depend on the object size. This was mostly due to the scatter characteristics of an MV beam. The reconstructed intensity for water changed depending on whether rFOV or eFOV mode was used during the acquisition. Hence, two different calibration curves were used in this study. Within the distinction of the imaging mode used, the size of the object also affected the image quality. Each protocol provided the option to choose from "small," "medium," and "large" settings, referring to the object size, to optimize the applied filters. To be consistent, only "medium" scan settings have been used in this study. However, the additional differentiation may improve image quality and consequently dose calculation accuracy, if found to be necessary. Furthermore, truncated anatomy caused missing data artifacts and altered the HU values, causing reduced image quality. Additionally, movement during the acquisition caused motion artifacts. Lastly, the position within the FOV can affect accurate image reconstruction and should be considered. The image reconstruction, which uses certain imaging filters to improve image quality, was optimized with the anatomy centered in the beam field. MV CBCT imaging with patients far off‐center may cause these to be less accurate, altering image intensity locally.

In addition to these considerations during patient setup, cone‐beam imaging may be restricted due to the limited FOV and, especially for extremities, clearance between the machine and the patient.

### E. Expected dose calculation accuracy for treatment site

To assess the described limitations and potentialities for MV CBCT‐based dose planning, the patient library was created considering a wide range of patient sites. Based on clinical experiences, treatment sites were categorized in four groups as follows and studied for dose planning: brain, thorax/spine, pelvis, and extremities.

Within the brain group, all treatments to the brain were predicted accurately using MV CBCT. The bone and soft tissue density were clearly identifiable on all brain scans. Nasal cavities caused the highest local dose differences within the dose calculation comparison.

The highest mean differences in dose predictions were within or beyond lung tissue in the thoracic spine group. The dose calculation accuracy largely depended on whether or not the beam traversed lung tissue. Motion due to breathing during the image acquisition caused streaking artifacts, which provided difficulties for correct intensity display. The static air inserts used during the reconstructed intensity‐to‐density calibration were not comparable to lung tissue in MV CBCT. Thus, the density of lung tissue varied significantly between patients, causing large local dose differences. Nevertheless, besides the general awareness of these discrepancies, they are not clinically relevant in palliative treatment situations. While it might be difficult to build an adequate phantom, a collection of patient images could be used to further assess the density for lung tissue and improve dose calculation predictions within lung tissue.

All pelvic images within the scope of this study were acceptable for treatment planning, provided that the patient was centered within the beam field during imaging. Difficulties for this group included truncation of the anatomy for patients exceeding the extended FOV. Capturing the entire anatomic site along the beam path was prerequisite for dose calculation and had to be considered during patient setup. In certain cases, only a single beam treatment was possible.

Extremities can often cause problems for machine clearance and patient safety. Although the rFOV was usually sufficient to image an arm, a knee, or a foot, clearance was difficult to achieve for a shoulder or a thigh, due to the target's off‐center position. For these cases, alternative CBCT protocols might be necessary to change the gantry start and end position to provide machine clearance. Due to a limited number of clinical cases for the duration of this study, the dosimetric evaluation for extremities will need further attention.

Despite local dose differences, the total number of MU per plan did not change significantly between dose calculations to the mid‐plane on CT and MV CBCT images. It shows the ability to use MV CBCT for simple dose planning. However, it is important to keep in mind which anatomical characteristics decrease the local dose calculation accuracy to prescribe to a point outside these areas.

### F. kV CBCT and MV CT imaging

Within the scope of this paper, only MV CBCT‐based treatment plan accuracy was assessed. Nevertheless, the workflow described here is adaptable to kV CBCT‐ and MV CT‐equipped linacs, which makes it versatile. kV CBCT image characteristics vary from MV CBCT images. Consequently, the feasibility for the treatment sites listed here would need to be verified separately through an additional dose comparison study.

A similar workflow as presented above has previously been described in the literature in combination with TomoTherapy (Accuray Corp., Sunnyvale, CA) and StatRT, commercial software specifically developed for TomoTherapy.^(10)^ Additional research for a similar procedure on TomoTherapy exists for the treatment of spine metastases.^19^, ^20^, ^21^, ^22^ Although StatRT offers advanced capabilities for urgent treatments, it is limited to TomoTherapy. The workflow described here is independent of commercial software, such as StatRT. It provides a technically advanced, fast, and safe option for machines using kV CBCT or MV CBCT on‐board imaging systems to perform urgent radiation therapy on different treatment sites, thus offering a unique alternative to palliative treatments on TomoTherapy.

## V. CONCLUSIONS

We have developed and implemented a clinical workflow to enable rapid and safe urgent treatments. All steps (CT simulation, dose planning, patient verification and quality assurance, and dose delivery) can be performed in one session with the patient on the linac couch. Dose calculation based on MV CBCT images can be used for a large number of palliative and emergency treatment cases. Sufficient dose precision was demonstrated for brain, pelvis, and extremity fields, as well as the accuracy of calculated MU for all thoracic spine treatments in palliative treatment situations. However, dose plan accuracy depends on the prescription point in these cases. A separate CBCT protocol and a thorax‐specific HU‐to‐density calibration curve for thoracic scans should be considered to improve dose calculations within and around lung tissue.

The clinical use of this technique for whole‐brain treatment is underway in our clinic. The results obtained in this study are promising to make MV CBCT‐based dose calculation possible for other emergency treatments, as well. Additional pelvic cases and extremity radiotherapy cases need to be studied to assess the full range of site‐specific planning accuracies before the clinical implementation of this technique. Once this has been established, a detailed workflow description will be written. Additional clinical experience will allow for a quantitative evaluation of the time, patient comfort, and dosimetric benefits that can be reached through this workflow.

## References

[1] Morin O and Pouliot J . Megavoltage cone‐beam IGRT. In: Bourland JD, editor. Image‐guided radiation therapy. Boca Raton (FL): CRC Press, Taylor & Francis Group; 2012.

[2] Chen J , Morin O , Aubin M , Bucci MK , Chuang CF , Pouliot J . Dose‐guided radiation therapy with megavoltage cone‐beam CT. Br J Radiol. 2006;79:S87–98. 16980688 10.1259/bjr/60612178

[3] Petit SF , van Elmpt WJ , Nijsten SM , Lambin P , Dekker AL . Calibration of megavoltage cone‐beam CT for radiotherapy dose calculations: correction of cupping artifacts and conversion of CT numbers to electron density. Med Phys. 2008;35(3):849–65. 18404922 10.1118/1.2836945

[4] Cheung J , Aubry JF , Yom SS , Gottschalk AR , Celi JC , Pouliot J . Dose recalculation and the dose‐guided radiation therapy (DGRT) process using megavoltage cone‐beam CT. Int J Radiat Oncol Biol Phys. 2009;74(2):583–92. 19345518 10.1016/j.ijrobp.2008.12.034

[5] Thomas TH , Devakumar D , Purnima S , Ravindran BP . The adaptation of megavoltage cone beam CT for use in standard radiotherapy treatment planning. Phys Med Biol. 2009;54(7):2067–77. 19287087 10.1088/0031-9155/54/7/014

[6] Hughes J , Holloway LC , Quinn A , Fielding A . An investigation into factors affecting electron density calibration for a megavoltage cone‐beam CT system. J Appl Clin Med Phys. 2012;13(5):93–107. Retrieved February 19, 2014 from http://www.jacmp.org 10.1120/jacmp.v13i5.3271 PMC5718240 22955638

[7] Morin O , Chen J , Aubin M , et al. Dose calculation using megavoltage cone‐beam CT. Int J Radiat Oncol Biol Phys. 2007;67(4):1201–10. 17336221 10.1016/j.ijrobp.2006.10.048

[8] Aubry JF , Cheung J , Morin O , Gottschalk A , Beaulieu L , Pouliot J . Correction of megavoltage cone‐beam CT images of the pelvic region based on phantom measurements for dose calculation purposes. J Appl Clin Med Phys. 2009;10(1):33–42. Retrieved March 21, 2012 from http://www.jacmp.org 10.1120/jacmp.v10i1.2852 PMC5720499 19223832

[9] Létourneau D , Wong R , Moseley D , et al. Online planning and delivery technique for radiotherapy of spinal metastases using cone‐beam CT: image quality and system performance. Int J Radiat Oncol Biol Phys. 2007;67(4):1229–37. 17336223 10.1016/j.ijrobp.2006.09.058

[10] McIntosh A , Dunlap N , Sheng K , et al. Helical tomotherapy‐based STAT RT: dosimetric evaluation for clinical implementation of a rapid radiation palliation program. Med Dosim. 2010;35(4):280–86. 19944589 10.1016/j.meddos.2009.09.002

[11] Faddegon BA , Wu V , Pouliot J , Gangadharan B , Bani‐Hashemi A . Low dose megavoltage cone beam computed tomography with an unflattened 4 MV beam from a carbon target. Med Phys. 2008;35(12):5777–86. 19175135 10.1118/1.3013571

[12] Beltran C , Lukose R , Gangadharan B , Bani‐Hashemi A , Faddegon BA . Image quality & dosimetric property of an investigational imaging beam line MV‐CBCT. J Appl Clin Med Phys. 2009;10(3):37–48. Retrieved February 19, 2014 from http://www.jacmp.org 10.1120/jacmp.v10i3.3023 PMC5720554 19692984

[13] Gayou O . Influence of acquisition parameters on MV‐CBCT image quality. J Appl Clin Med Phys. 2012;13(1):14–26. Retrieved January 13, 2015 from http://www.jacmp.org 10.1120/jacmp.v13i1.3638 PMC5716124 22231215

[14] Langen KM , Meeks SL , Poole DO , et al. The use of megavoltage CT (MVCT) images for dose recomputations. Phys Med Biol. 2005;50(18):4259–76. 16148392 10.1088/0031-9155/50/18/002

[15] Aubry JF , Pouliot J , Beaulieu L . Correction of megavoltage cone‐beam CT images for dose calculation in the head and neck region. Med Phys. 2008;35(3):900–07. 18404926 10.1118/1.2839146

[16] Morin O , Aubry JF , Aubin M , et al. Physical performance and image optimization of megavoltage cone‐beam CT. Med Phys. 2009;36(4):1421–32. 19472649 10.1118/1.3096706

[17] Battista JJ and Bronskill MJ . Compton scatter imaging of transverse sections: an overall appraisal and evaluation for radiotherapy planning. Phys Med Biol. 1981;26(1):81–99. 7243873 10.1088/0031-9155/26/1/009

[18] Rogers M , Kerr A , Salomons G , Schreiner LJ . Quantitative investigation of megavoltage computed tomography [proceedings paper]. In: Flynn MJ, editor. Medical Imaging 2005: Physics of medical imaging. Bellingham (WA): SPIE; 2005 p. 685–94.

[19] Mahan SL , Ramsey CR , Scaperoth DD , Chase DJ , Byrne TE . Evaluation of image‐guided helical tomotherapy for retreatment of spinal metastasis. Int J Radiat Oncol Biol Phys. 2005;63(5):1576–83. 16125871 10.1016/j.ijrobp.2005.05.015

[20] Kim B , Soisson ET , Duma C , et al. Image‐guided helical tomotherapy for treatment of spine tumors. Clin Neurol Neurosurg. 2008;110(4):357–62. 18295971 10.1016/j.clineuro.2007.12.024

[21] MacPherson M , Montgomery L , Fox G , et al. On‐line rapid palliation using helical tomotherapy: a prospective feasibility study. Radiother Oncol. 2008;87(1):116–18. 18329119 10.1016/j.radonc.2008.01.017

[22] Rong Y , Yadav P , Paliwal B , Shang L , Welsh JS . A planning study for palliative spine treatment using StatRT and megavoltage simulation. J Appl Clin Med Phys. 2011;12(1):97–107. 10.1120/jacmp.v12i1.3348 PMC5718582 21330983

